# Use of Alternative Gelling Agents Reveals the Role of Rhamnolipids in *Pseudomonas aeruginosa* Surface Motility

**DOI:** 10.3390/biom11101468

**Published:** 2021-10-06

**Authors:** Charles D. Morin, Eric Déziel

**Affiliations:** Centre Armand-Frappier Santé Biotechnologie, Institut National de la Recherche Scientifique (INRS), Laval, QC H7V 1B7, Canada; charles.morin@inrs.ca

**Keywords:** gellan gum, carrageenan, swarming motility, wetting agents, bacterial behaviour

## Abstract

*Pseudomonas aeruginosa* is a motile bacterium able to exhibit a social surface behaviour known as swarming motility. Swarming requires the polar flagellum of *P. aeruginosa* as well as the secretion of wetting agents to ease the spread across the surface. However, our knowledge on swarming is limited to observed phenotypes on agar-solidified media. To study the surface behaviour and the impact of wetting agents of *P. aeruginosa* on other surfaces, we assessed surface motility capabilities of the prototypical strain PA14 on semi-solid media solidified with alternative gelling agents, gellan gum and carrageenan. We found that, on these alternative surfaces, the characteristic dendritic spreading pattern of *P. aeruginosa* is drastically altered. One striking feature is the loss of dependence on rhamnolipids to spread effectively on plates solidified with these alternative gelling agents. Indeed, a *rhlA*-null mutant unable to produce its wetting agents still spreads effectively, albeit in a circular shape on both the gellan gum- and carrageenan-based media. Our data indicate that rhamnolipids do not have such a crucial role in achieving surface colonization of non-agar plates, suggesting a strong dependence on the physical properties of the tested surface. The use of alternative gelling agent provides new means to reveal unknown features of bacterial surface behaviour.

## 1. Introduction

*Pseudomonas aeruginosa* is a versatile bacterium—mainly recognized as an important opportunistic pathogen—capable of complex social behaviours such as biofilm formation and surface group motility [1]. Multiple types of bacterial motilities were originally described by Jorgen Henrichsen [2]. Of those, swimming, swarming, twitching and sliding have been reported in *P. aeruginosa*. Swimming is a single-cell, flagellum-driven motility that occurs in liquid environments and through soft gels (i.e., up to 0.3% agar). Swarming motility, on the other hand, is a social form of flagellum-dependant translocation on semi-solid surfaces that requires the secretion of wetting agents to facilitate the movement of the bacteria over the surface [3]. This coordinated population movement often gives rise to complex dispersion patterns, such as the characteristic “concentric circle” shape formed by *Proteus mirabilis* [4], or the fractal patterns of *Bacillus subtilis* [5]. The surfactants produced during such surface motility are typically amphiphilic molecules such as lipopeptides (e.g., surfactin for *Bacillus*) or glycolipids (e.g., rubiwettins for *Serratia rubidaea*) [6,7]. In the case of *P. aeruginosa*, these surfactants are 3-(3-hydroxyalkanoyloxy)alkanoic acids (HAA), monorhamnolipids and dirhamnolipids sequentially produced by the RhlA, RhlB and RhlC enzymes, respectively [8]. HAAs and rhamnolipids not only promote bacteria spreading across the surface during swarming motility, but also induce the characteristic dendritic swarm pattern often displayed by *P. aeruginosa* [9,10]. As HAAs do not possess rhamnose moieties, they are more hydrophobic than mono- and dirhamnolipids. Because of this chemical property, they also diffuse less in the swarming medium, compared to its rhamnose-bearing counterparts, creating an increasing gradient ratio away from the colony [9]. The production of HAAs and rhamnolipids is controlled at the transcriptional level by the quorum sensing regulator RhlR once the bacterial population has reached a threshold density [11]. Along with rapid surface colonization, swarming has been linked to increased antibiotic tolerance [12,13]. Twitching is another form of social motility, where cells drag themselves across a solid surface (such as polystyrene) through the action of their type IV pili (T4P) [14]. Sliding is a surfactant-driven form of motility that was observed only in the absence of functional flagellum and T4P [15]. Another type of surface motility more recently described for *P. aeruginosa* and other bacterial species is surfing [16,17]. This motility is flagellum-driven, it does not require the production of wetting agents but instead exploits the wetting properties of mucin supplemented to the culture medium.

Agar is the most used gelling agent in routine lab experiments. Most of our knowledge on bacterial behaviours on semi-solid and solid gels comes from observations of agar-based plate assays. Agar is primarily formed by chains of disaccharide agarose (formed by units of d-galactose and l-galactopyranose), produced by some red algae species [18]. Alternatives to agar, such as carrageenan and gellan gum, have gained popularity in recent years due to their lower cost and different gelling properties [19]. Carrageenan is also obtained from red algae and is formed of chains of d-galactopyranose with varying numbers of lateral sulphate groups [20]. It possesses a greater tolerance to high pH than agar [21]. Gellan gum is formed by chains of tetrasaccharides (d-glucose, d-glucoronic acid, d-glucose, l-rhamnose). This polysaccharide is produced from *Sphingomonas* species [22]. Its higher melting point makes it suitable for the culture of thermophiles [23].

A phenotype such as swarming motility is extremely sensitive to culture conditions, it is, therefore, challenging to obtain reproducible data [24]. Indeed, this motility phenotype is directly linked to the characteristics of the agar gel surface, such as the gelling agent concentration, drying time, osmotic pressure and wettability [25,26]. Seemingly trivial factors such as the brand of agar were reported to influence the swarming phenotype [27,28,29]. Here, we investigated the effect of two alternative gelling agents—carrageenan and gellan gum—on surface spreading of *P. aeruginosa*. Using standard swarming conditions, we reveal novel motility phenotypes that diverge from standard swarming motility on agar gels, highlighting our very limited understanding of bacterial social surface motility phenomena.

## 2. Materials and Methods

### 2.1. Bacterial Strains and Culture

Bacterial strains used in this study are listed in Table 1. Bacteria were routinely cultured in Tryptic Soy Broth (TSB, Alpha Biosciences, Baltimore, MD, USA) at 37 °C in a roller drum, or on Tryptic Soy Agar (TSA, Alpha Biosciences) plates. Swarming medium was based on modified M9 with dextrose and casamino acids (M9DCAA), as described [25]. Briefly, M9DCAA medium (20 mM NH_4_Cl, 12 mM Na_2_HPO_4_, 22 mM K_2_HPO_4_, 8.6 mM NaCl, 0.5% casamino acids (BD Difco, Franklin Lakes, NJ, USA), 1 mM CaCl_2_, 1 mM MgSO_4_, 11.1 mM dextrose) was solidified with either 0.5% Bacto™ Agar (BD Difco), 0.5% carrageenan (mixture of κ-carrageenan and λ-carrageenan, Millipore Sigma, Burlington, MA, USA) or 0.1% gellan gum (Phytagel™, Millipore Sigma) to obtain a semi-solid gel. A volume of 20 mL of this medium was poured into 100 mm polystyrene Petri dishes and left to dry under the flow of a biosafety cabinet for 25 min.

### 2.2. Swarming Motility Assay

Overnight, bacterial cultures were diluted 1/30 in TSB and incubated at 37 °C for about 4 h to late exponential phase. All the cultures were adjusted to an OD_600_ of 3.0 and 5 µL of each were spot-inoculated onto the center of one semi-solid M9DCAA plate. The plates were incubated at 30 °C overnight (between 16 and 20 h). After incubation, a picture of each whole plate was taken with a Panasonic Lumix DMC-ZS60 digital camera in a custom-made lightbox. Higher magnification images were captured using an Olympus SZX16 stereomicroscope. Each combination of mutants and gelling agents was tested in triplicates at least three times.

### 2.3. Construction of ΔfliC Mutant

Deletion of the *fliC* gene was achieved by allelic exchange using suicide vector pEX18Gm-Δ*fliC* using standard procedure. Briefly, pEX18Gm vector was transferred into *P. aeruginosa* via mating with the *E. coli* donor strain: both donor strain and recipient strains were grown in TSB without shaking at 37 °C and 42 °C, respectively. After a few hours of growth, cells were centrifuged at 10,000× *g* for 1 min, supernatant was discarded and cells were mixed into 100 µL TSB. This cell suspension was dropped onto a Tryptic Soy Agar (TSA, Alpha Biosciences) plate and left overnight at 37 °C. Bacteria were scraped from the surface of the agar gel, suspended into buffered saline, and diluted prior to spread on selective medium containing 15 µg/mL gentamycin to select for recipient merodiploid cells that integrated the plasmid and 25 µg/mL triclosan to counter-select the donor cells. Subsequent selection of the second recombination event was achieved by plating onto tryptone yeast medium supplemented with 15% sucrose [33]. Loss of flagellum was confirmed using a swimming assay [34].

### 2.4. Construction of rhlC- Mutant

The *rhlC* gene sequence was amplified from the PA14 genome using primers *rhlC*_fwd (CGCTGGCGCCAAGCTTATGGACCGGATAGACATGG) and *rhlC*_rev (CGAAGCTAGCGAATTCGTACAGGGGCACGTCCAG). This sequence contains an intrinsic PstI restriction site and was cloned into plasmid pNTPS138 to produce pNTPS183-*rhlC*. The sequence of tetracycline resistance cassette (*tet*^R^) was amplified from plasmid pACYC184 using primers *tet*_fwd (AAAACTGCAGAGCAAGAGATTACGCGCAGA) and *tet*_rev (AAAACTGCAGTTTGCGCATTCACAGTTCTC), both containing PstI restriction sites. The *tet*^R^ gene sequence was then cloned into the PstI site of *rhlC* gene sequence of pNTPS138-*rhlC.* The resulting plasmid pNTPS138-*rhlC*::*tet*^R^ was transformed into the conjugative *E. coli* strain SM10. The plasmid was transferred into the recipient *P. aeruginosa* wild-type strain using bacterial conjugation as described above. Recipient cells that integrated the resistance cassette into their *rhlC* gene were selected on medium containing 125 µg/mL tetracycline and were confirmed by PCR and sequencing.

## 3. Results and Discussion

### 3.1. Surface Motility Pattern Is Affected by the Type of Gelling Agent Used

**Figure 1:**
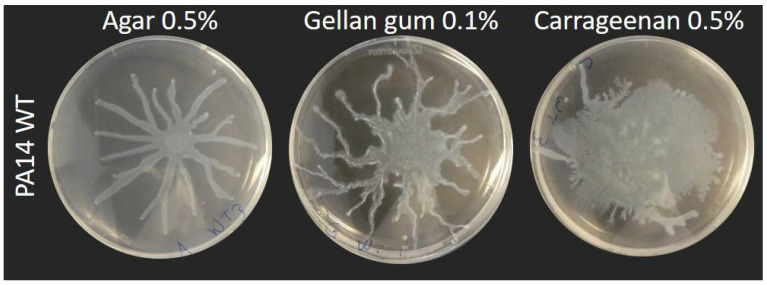
Social surface motility of *P. aeruginosa* PA14 WT on medium solidified with various gelling agents. Swarming medium M9DCAA was solidified with 0.5% agar, 0.1% gellan gum or 0.5% carrageenan. Each picture is representative of three biological replicates.

Our current definition of swarming motility is based on the use of agar as a culture medium solidifying agent. To explore the impact of gel properties on the spreading phenotype of *P. aeruginosa*, we used alternative gelling agents, gellan gum and carrageenan, to solidify the M9DCAA medium routinely used for swarming motility assays. We tested various percentages of gelling agents: 0.1%, 0.3%, 0.5% and 1% for gellan gum and 0.5%, 0.7% and 1% for carrageenan. Figure S1 shows the spreading phenotype of *P. aeruginosa* PA14 WT at 0.5% and 0.7% for agar, 0.5% and 0.7% for carrageenan and 0.1% and 0.3% for gellan gum. Under our conditions, concentrations that allowed the surface spreading of *P. aeruginosa* were 0.1% for gellan gum and 0.5% for carrageenan (Figure 1 and Figure S1). We also tested xanthan gum but were unable to form stable gels, even at concentrations up to 2% (data not shown).

Swarming motility of wild-type *P. aeruginosa* strains, such as strain PA14, typically forms a characteristic dendritic pattern with tendrils spreading away from the inoculation center and from each other (e.g., Figure 1). When we solidified our M9DCAA medium with either 0.5% carrageenan or 0.1% gellan gum, we noticed an impact on the dissemination of *P. aeruginosa* during surface motility. With gellan gum, the motility pattern displayed finer tendrils which spread away from the center, in a manner similar to agar gels. With carrageenan, the spread pattern was circular and more disorganized, with only a few occasional tendrils showing per plates (Figure 1), although we found this phenotype to be inconsistent (i.e., sometimes there were lots of tendrils or none at all). Figure S2 visually presents this inconsistency from a sample of assays of PA14 WT on carrageenan. In these conditions, there seems to be a fine balance between a branched pattern and a circular pattern. All in all, this suggested that classic tendril-like swarming is possible on those alternative surfaces. Multiple factors influence the formation of a branching swarming pattern, such as volumetric growth, chemotaxis and—especially in the case of *P. aeruginosa*—the presence of surface tension gradients induced by the release of wetting agents [35,36,37]. *P. aeruginosa* produces HAA and rhamnolipids as its surfactant, which play a role in the outcome of the swarm pattern [9,10]. We hypothesized that rhamnolipids would likely play an important role in the slightly different swarm patterns displayed on carrageenan and gellan gum. We then set out to test the motility of three mutants on these different gels: *rhlA*- (unable to produce any surfactant), *rhlB*- (producing only HAA) and *rhlC*- (producing HAA and mono-rhamnolipids) [8].

### 3.2. Rhamnolipids Are Not Required for Motility on Alternative Gelling Agents but Still Contribute to the Motility Pattern

**Figure 2:**
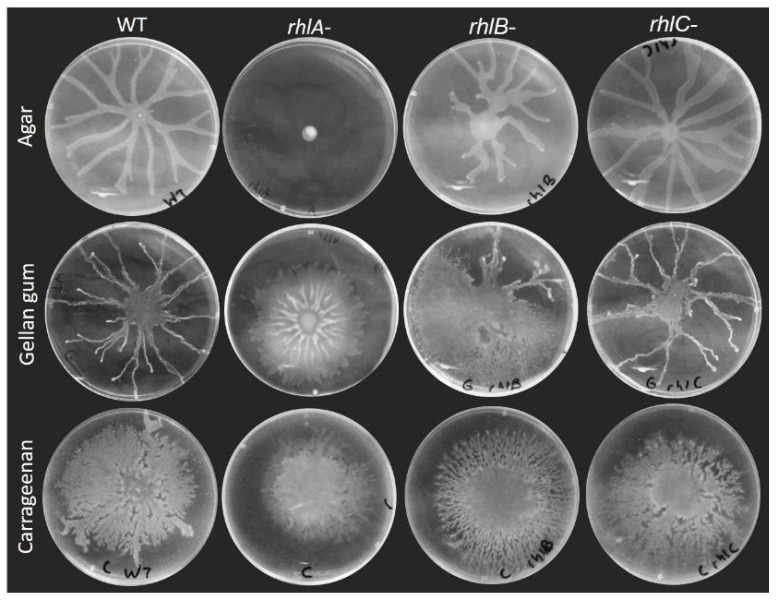
Surface motility of rhamnolipids biosynthesis mutants (*rhlA*-, *rhlB*- and *rhlC*-) compared to WT. Motility assays were conducted on M9DCAA solidified with either agar (0.5%), carrageenan (0.5%) or gellan gum (0.1%). Pictures are representative of at least three biological replicates, repeated three times.

**Figure 3:**
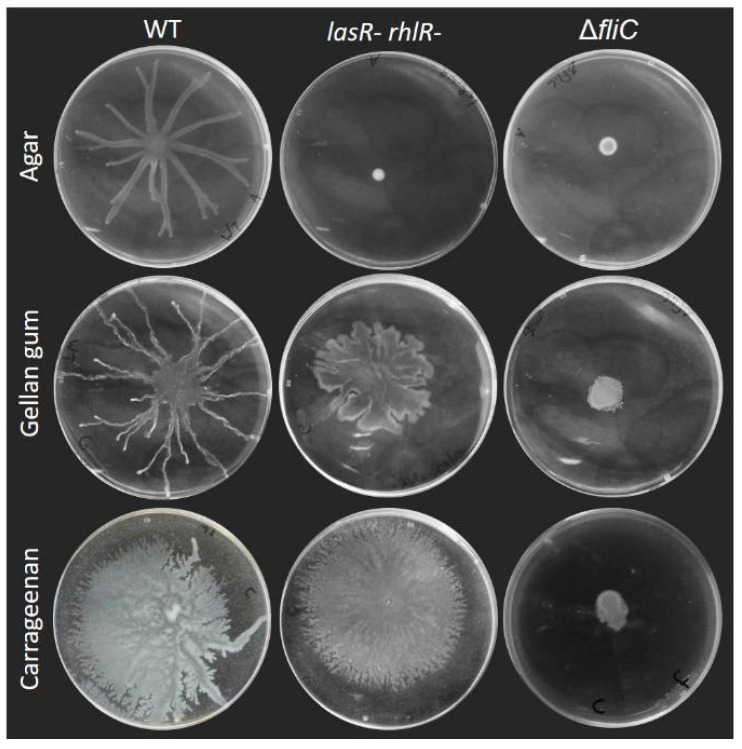
Surface motility on carrageenan and gellan gum media does not require quorum sensing and depends on the flagellum. Swarming motility of quorum sensing-deficient *lasR*- *rhlR*- double mutant and flagellum-deficient *fliC*- of *P. aeruginosa* PA14 compared to wild-type (WT) on agar M9DCAA semi-solid gels with agar (0.5%), carrageenan (0.5%) and gellan gum (0.1%). Each picture is representative of three biological replicates.

Swarming motility of *P. aeruginosa* is considered to require both a functional flagellum to allow movement on the surface and the production of a wetting agent to reduce surface tension. This is true on agar gels, as Figure 2 shows the loss of swarming of a *rhlA*- mutant (Figure 3 shows the swarming-deficient phenotype of a *fliC*- mutant). As we previously reported [9], HAA will suffice to promote surface motility: a *rhlB*- mutant will swarm almost as well as the WT (with a slightly altered pattern) and a *rhlC-* mutant is nearly identical to the WT (Figure 2).

Surprisingly, however, this was not true when swarming was assessed on plates solidified with the other gelling agents. A *rhlA*- mutant retained its ability to spread across the surface of a carrageenan or gellan gum gel, albeit in a circular form that is distinct between the two agents. This suggests that the need to produce a wetting agent is not indispensable for swarming motility, or that the surface spreading phenotype we observe is not swarming *per se*. Interestingly, the colony pattern of the *rhlB*- mutant was distinct from the *rhlA*- and the WT, while *rhlC*- was similar to the WT under all conditions tested. Therefore, it appears that HAA, mono-rhamnolipids and di-rhamnolipids would each contribute differently to the surface group behaviour pattern of *P. aeruginosa*.

**Figure 4:**
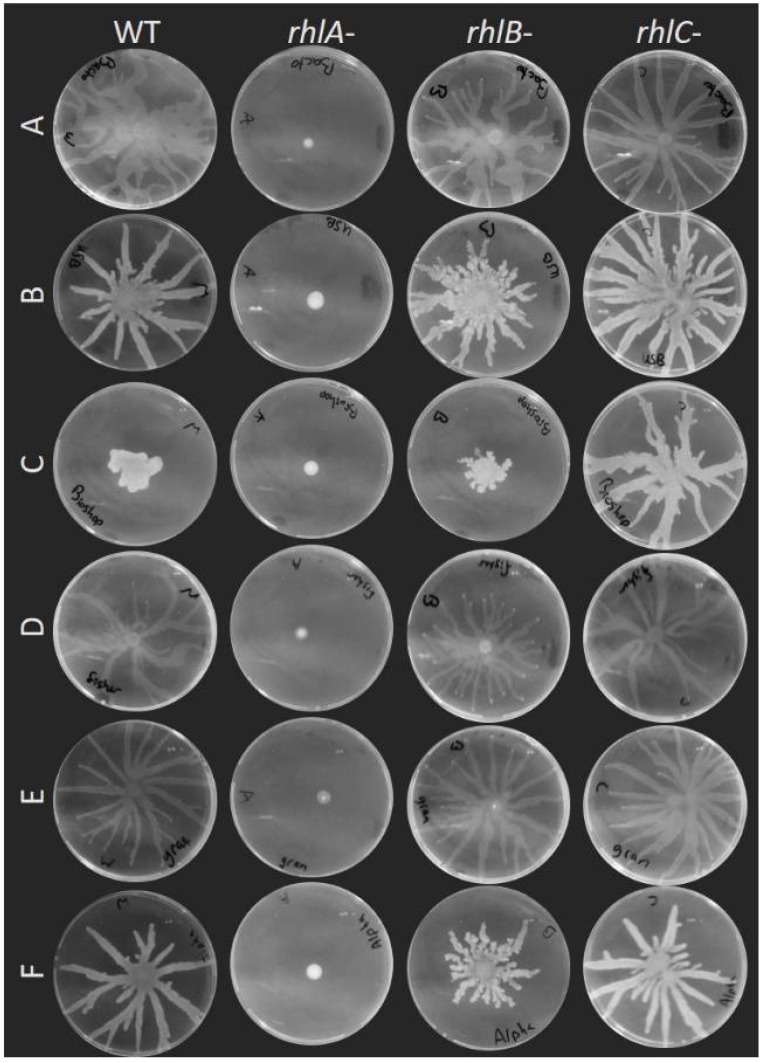
Swarming assay of PA14 WT, *rhlA*-, *rhlB*- and *rhlC*- mutants on M9DCAA media solidified with different agar brands. A, Bacto™ Agar (BD Difco). B, Agar, bacteriological (USB, Salem, MA, USA). C, Agar, bacteriological grade (BioShop, Burlington, ON, Canada). D, Agar (Fisher, Waltham, MA, USA). E, Granulated agar (BD Difco). F, Agar, bacteriological (Alpha Biosciences). Each image is representative of three biological replicates.

Swarming motility was already reported to be affected by the brand of agar used to conduct the assays. Traces of furan-2-carboxylic acid were found to reduce the spread of swarming colonies of *Pseudomonas colierea*; this molecule was present in variable quantities between different agar brands [27]. Certain agar brands used for *E. coli* and *Salmonella typhimurium* did not allow for a strong swarming motility phenotype compared to others [28]. For *P. aeruginosa*, the use of different agar brands caused alteration in the swarming pattern [29,38]. We asked ourselves if the consensus that rhamnolipids are essential for swarming on agar still holds true on various agar brands. We assessed the swarming phenotype of PA14 *rhlA-*, *rhlB-* and *rhlC-*, along with the wild-type, on these media. Figure 4 shows that none of the different agar brands allowed for the spread of the surfactant-null *rhlA*- mutant. Furthermore, the loss of either *rhlB* or *rhlC* had a similar impact on the swarming on various agar gels, despite altered phenotypes of the wild-type. We conclude that changing the type of gelling agent altersmore drastically the motility phenotype of *P. aeruginosa* than the brand. Still, the brand of agar clearly affects the swarming patterns, and this should be considered when comparing independent studies.

There is a precedent for such RhlA-independent surface motility: *P. aeruginosa* surfing motility was characterized as a flagellum-dependent motility occuring on culture medium supplemented with mucins, highly O-glycosylated proteins produced by epithelial tissues. Under these conditions, *P. aeruginosa* spread in a circular pattern independent of the production of rhamnolipids [16]. This phenotype was proposed to be due to the lubricant action of mucin since the addition of the surfactant TWEEN 20 had a similar effect. To determine if motility of the *rhlA*- mutant presented here consisted of surfing, we tested the surface motility of a double *lasR*- *rhlR*- mutant on gellan and carrageenan (Figure 3). The loss of these two genes—which are required for surfing [16]—did not prevent the surface spreading under our conditions, indicating that surfing does not explain the phenotype we observed.

**Figure 5:**
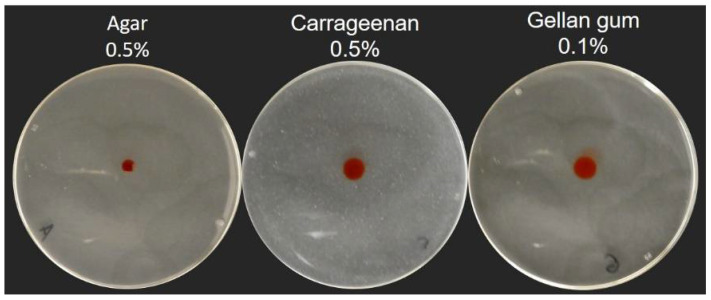
Spreading of aqueous coloring agent drops onto swarming gels. A 5 µL drop of 1% Red Congo spreads more when spotted onto a carrageenan or gellan gum gels, compared to an agar gel. Pictures were taken five minutes after the drop was added.

One explanation would be that gels produced with gellan gum and carrageenan possess different surface tensions compared to agar. Indeed, we routinely noticed that drops of bacterial suspension spread a lot more on gellan gum and carrageenan gels compared to agar after inoculation, in the same manner that Figure 5 shows the spread of a drop of colored water on each gel surface. This spreading likely also explains the differences in colony sizes of the Δ*fliC* mutant between agar and the other gelling agents (Figure 3). Such differences in surface properties, or the addition of an external wetting agent (mucin or surfactant), could explain the ability to spread across surfaces without the need to release a wetting agent such as rhamnolipids. Studies have reported that the addition of surfactants into the swarming agar affects surface motility on agar [16,26].

**Figure 6:**
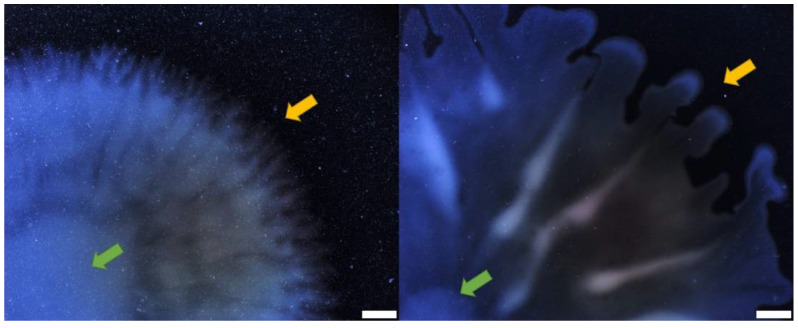
Darkfield images of colony border of PA14 *rhlA*- mutant on alternative gelling agents. Carrageenan 0.5% (**left**) and gellan gum 0.1% (**right**). Yellow arrows point towards colony border, green arrows point towards colony center. Images were taken with Olympus SZX16 stereomicroscope. White scale bars represent 2 mm.

Multiple forces have been described as important factors to induce branching instabilities and finger-like colony morphologies during bacterial swarming colony growth. Volumetric expansion relies on the mass buildup to push down on the colony, causing a passive expansion that can lead to an uneven distribution of bacteria and the formation of finger-like protrusion [35]. In a similar manner, chemotactic expansion relies on the detection of nutrients available on the vicinity and the diffusion of these nutrients that will lead to the formation of a dendritic pattern [35]. Swarming of *P. aeruginosa* is considered to not require chemotaxis; a transcriptomic analysis of swarm population indicated that chemotaxis genes were downregulated [39,40]. Additionally, *P. aeruginosa* tendrils can move towards nutrient-depleted zones [37]. For biosurfactant-producing bacterial species, such as *P. aeruginosa*, tensioactive molecules are also involved in shaping the colony in a branched pattern as the result of surface tension gradients induced by the surfactants [10,36,37]. The latter mechanism is the most important in dictating the swarming morphology of *P. aeruginosa*: we showed that HAA and rhamnolipids contributed to tendril formation by a combined effect of attraction and repulsion [9]. Indeed, the presence of HAA repels incoming tendrils, while di-rhamnolipids attracts them. Thus, it makes sense that *rhlA*- mutants, if allowed to spread, would do so in a circular pattern. However, spreading patterns between a *rhlA*- mutant on gellan gum was still slightly different from the one on carrageenan. The former had a flower-shaped pattern with a smooth edge while the latter displayed tiny tendrils on its edges (Figure 6). This is interesting since, apart from rhamnolipids, no other factors are known to induce tendrils in *P. aeruginosa*. Using alternative gelling agents such as carrageenan might allow us to discover another mechanism that coordinates the colony expansion of *P. aeruginosa* during surface motility.

Another type of motility called sliding has been reported in *P. aeruginosa*, but this motility requires the production of a surfactant and does not require the flagellum [41]. Since surface motility was essentially abolished in the Δ*fliC* mutants for all gelling agents tested, this indicates that all forms of spread in these conditions depended on the flagellum. This suggests that the motility highlighted here was not sliding. Additionally, for media solidified by carrageenan and gellan gum, the production of a wetting agent was no longer necessary. In the literature, swarming is defined as a social surface motility requiring a functional flagellum and the production of a wetting agent [3]. Thus, the question remains: should these surface motilities be categorized as swarming in conditions where alleviating the surface tension is not required?

Apart from the impact of the gelling agent that we underlined here, medium composition was reported to impact the surface motility behaviour of *P. aeruginosa*. The contribution to the swarming of multiple two-components systems is dependent on medium composition [42]. Both *lasRrhlR* and *rhlAB* double mutants were able to spread in a similar fashion to the WT on FAB medium with glutamate, as opposed to the same medium with glucose or succinate [43]. Surface hardness and the osmolarity of the gel are two additional parameters that were reported to affect the swarming motility of *P. aeruginosa* [26,44]. Both parameters are tied to the medium composition: gel properties of carrageenan and gellan gum change depending on the pH and salt concentrations of the medium [21,22]; osmolarity was dependent on the concentration of soluble molecules (salts, nutrients) in the gel [26]. Together, these parameters interact with each other and influenc the surface behaviour of *P. aeruginosa*.

Agar was first used for the fabrication of culture media in 1882. Since then, it has become the primary gelling agent used in routine microbiology research [19]. Most of our knowledge on bacterial behaviour comes from assays conducted on agar-based media. Alternatives to agar as the main gelling agent have been increasingly popular over the last decades, mainly for the purpose of isolating new bacterial species [45,46,47,48,49,50]. However, very few reports discuss the effect of alternative gelling agents on microbial surface behaviours. Gellan gum was found to more rapidly induce the differentiation of cyanobacterial *Nostoc* species into their motile hormogonium state [51]. Some isolates from the Siberian soil were more motile on plates solidified with gellan gum [45]. Carrageenan was used to highlight the peculiar, snake-like spread of *Vibrio alginolyticus* [52]. Oftentimes, the motile phenotype described in these reports was “swarming in the broad sense” described by Henrichsen, i.e., any form of surface spreading [2]. Here, we show that changing the gelling agent had a pronounced impact on the surface motility behaviour displayed by *P. aeruginosa*.

## 4. Conclusions

To explore the impact of the wetting agents produced by *P. aeruginosa* on swarming motility, we used alternative gelling agents—gellan gum and carrageenan—instead of agar, to solidify our swarming medium. We found that simply changing the gelling agent had a profound impact on the surface spreading pattern of *P. aeruginosa*. Although all forms of surface motility appeared dependant on the presence of a functional flagellum, the loss of the wetting agent rhamnolipids did not impact these motile phenotypes in the same way as swarming. Therefore, we should always consider the gelling agent used as part of the factors to be considered when investigating bacterial social surface phenotypes and the impact of exoproducts on microbial behaviour. Maybe we need to revise our definition of swarming motility to consider conditions where the production of a wetting agent is not required.

## Figures and Tables

**Table 1:** Bacterial strains used in this study.

Strain	Stock #	Genotype	Reference
***Pseudomonas aeruginosa***			
PA14 WT	ED14	UCBPP-PA14, parental wild-type strain	[30]
PA14 *rhlA*-	ED1	PA14 *rhlA*::Mar2xT7	[31]
PA14 Δ*fliC*	ED3956	PA14 Δ*fliC*	This study
PA14 *rhlB*-	ED286	PA14 *rhlB*::Mar2xT7	[31]
PA14 *rhlC*-	ED777	PA14 *rhlC*::*tet*^R^	This study
PA14 *lasR*- *rhlR*-	ED266	PA14 *lasR*::Gm^R^ *rhlR*::*tet*^R^	[32]
***Escherichia coli***			
S17.1 (pEX18Gm-Δ*fliC*)	ED3955	*E. coli* strain with suicide vector pEX18Gm-Δ*fliC* for allelic exchange deletion of *fliC*	[15]
SM10 (pNTPS138-*rhlC*::*tet*R)	ED1271	*E coli* strain with suicide vector to insert a tetracycline resistance cassette into *rhlC* by allelic exchange.	This study

## Data Availability

Not applicable.

## Supplementary Materials

The following are available online at https://www.mdpi.com/article/10.3390/biom11101468/s1, Figure S1: Gelling agent percentage allowing for surface spreading of *P. aeruginosa* PA14 WT, Figure S2: Swarming phenotype of PA14 WT on carrageenan 0.5%.
