# Supplementary material for: Use of Alternative Gelling Agents Reveals the Role of Rhamnolipids in Pseudomonas aeruginosa Surface Motility
Source: Biomolecules. 2021 Oct 6;11(10):1468. doi: 10.3390/biom11101468 (PMC8533327; doi:10.3390/biom11101468)
Supplement: Supplementary file 1 [file biomolecules-11-01468-s001.zip › biomolecules-1338387-supplementary.pdf]

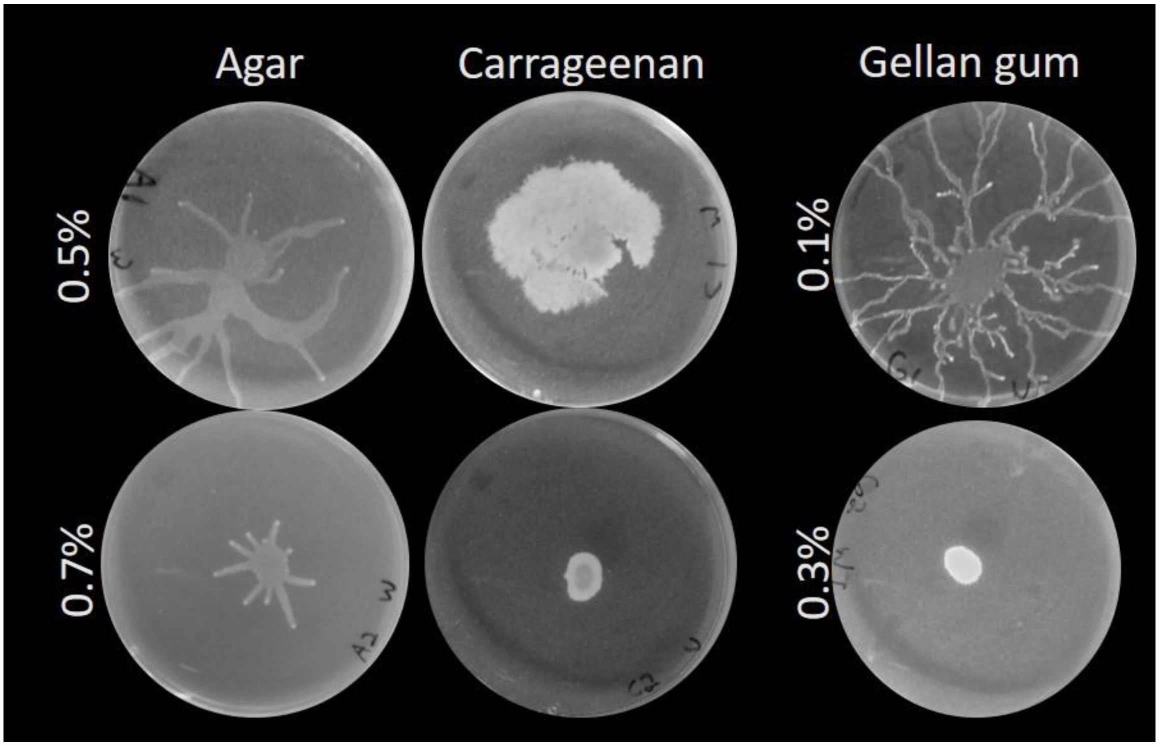

Figure S1. Gelling agent percentage allowing for surface spreading of *P. aeruginosa* PA14 WT. Concentration of 0.5% allows motility for agar and carrageenan, but not 0.7%. Concentration of 0.1% allows motility of PA14 WT for gellan gum, but not 0.3%

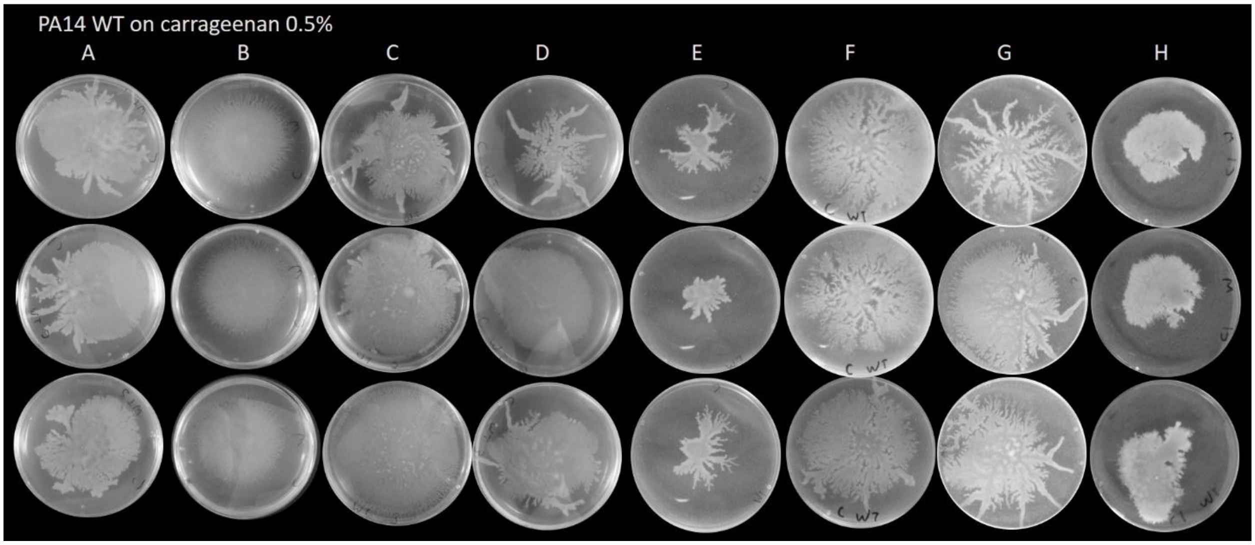

Figure S2. Swarming phenotype of PA14 WT on carrageenan 0.5%. Each letter presents the triplicate of PA14 WT from an independent assay.
